# Influence of saturation level on the acoustic emission characteristics of gas hydrate-bearing coal

**DOI:** 10.1038/s41598-024-57178-9

**Published:** 2024-03-16

**Authors:** Kang Yu, Yu Miao, Zhu Jinchao

**Affiliations:** School of Safety Engineering, Heilongjiang University of Science and Technology, Harbin, 150022 Heilongjiang China

**Keywords:** Coal and gas outburst, Coal mine gas prevention based on hydrate, Gas hydrate bearing coal, Acoustic emission characteristics, Saturation, Coal, Chemical engineering

## Abstract

To study the effects of gas hydrates on the prevention and control of coal and gas protrusions, this paper reports the results of acoustic emission experiments on coal bodies containing gas hydrates with different saturation levels. The results showed that few acoustic emission events were generated in the elasticity stages of coal bodies containing gas hydrates, and the first sudden increase in the number of ringing counts generally occurred before and after the yielding point. Additionally, the acoustic emission events in the yielding stage were more active, and the cumulative number of ringing counts increased faster. The peak ringing counts appeared around the damage point, and a small number of acoustic emission events were still generated after destruction of the coal samples. The cumulative ringing counts decreased linearly with increasing saturation. The effect of saturation on the cumulative ringing counts in the elasticity stage of the gas hydrate-containing coal samples was small, but the difference between the cumulative ringing counts in the yielding stage and those in the destruction stage was larger. The total cumulative ringing counts and the cumulative ringing counts during each stage for the gas hydrate-containing coal samples decreased with increasing enclosure pressure. The energy and amplitude of the loading process were consistent with the trend for the ringing counts. The acoustic emission ringing counts of gas-containing coals were greater than those of gas hydrate-containing coals in the yielding and destructing stages.

## Introduction

Coal is an important energy source for China's economy and is widely used in major basic industries such as power generation and smelting. In recent years, with increases in the mining intensity and demand, the depth of coal mining has increased at a rate of 8–12 m per year^1^. The phenomenon of high ground stress is prominent, and dynamic disasters such as coal and gas outbursts occur frequently; these disasters have a considerable impact on safe and efficient mining of the coal mines and pose a great threat to the safety of mine workers^2,3^. Mining is one of the main natural disasters faced, and in-depth research on coal and gas outburst prevention and control technologies is urgently needed.

In 2005, Wu et al.^4^ proposed a new way to prevent and control coal and gas outbursts by utilizing the conditions of mild gas hydrate generation, strong gas storage capacity, and slow decomposition. The main technical idea was to inject high-pressure water containing promoters into the coal seam, causing the gas and water to react to form solid hydrates, transforming gases into solid hydrates, and reducing the pressure of the coal seam gas. In addition, the improved mechanical properties of coal containing gas hydrates compared to those of coal containing gases has achieved the goal of preventing coal and gas outbursts^5–7^. The key to preventing coal and gas outbursts with hydrate technology lies in understanding the mechanical properties of the coal containing gas hydrates, the impact of gas hydrate generation on coal permeability, and the mechanism for gas hydrate generation. In the present research, it was found that the generation of gas hydrates changed the strength of the coal and reduced the permeabilities of the coal seams. As the saturation of gas hydrates gradually increased, this phenomenon becomes increasingly significant^8–13^. In the process of studying the mechanical properties of coal bodies containing gas hydrates, the coal and rock masses often experienced stress failure. During the process of load failure, the energy accumulated inside the coal and rock mass was released in the form of acoustic emission signals. Therefore, the internal state of the coal body can be determined directly from the acoustic emission signals^14^.

At present, research on acoustic emissions has focused mainly on coal or gas-bearing coal rock masses. Zhang et al.^15^ classified acoustic emission events into three bond classes, short, medium, and long, and proposed that these three bond classes reflected local damage, overall damage, and random damage processes, respectively. Xiao et al.^16^ reported that the acoustic emission activity gradually weakens with increasing shear angles. Zhang^17^ compared and analysed the evolution law of acoustic emission signals during the deformation and failure of raw coal and briquette and analysed the corresponding mechanisms. Liu et al.^18^ used statistical physics to study the acoustic emission signals of composite rocks. Song et al.^19^ studied the anisotropy of coal acoustic emission signals under uniaxial conditions. Wang et al.^20^ reported that acoustic emission mainly originated from microcrack activity in the process zone. Lu et al.^21^ reported that the acoustic emission b value fluctuated sharply before failure, indicating the formation of multiscale cracks inside the coal body. Yang et al.^22^ reported that the maximum acoustic emission energy occurred when the overall instability of the roof coal pillar bottom plate occurred at different height ratios. Mu et al.^23^ reported that the joint angle exhibited differentiated prepeak and postpeak acoustic emission characteristics with a 45° boundary. Meng et al.^24–26^ reported that the mechanical and acoustic emissions of coal samples with cement contents of 20% under confining pressures of 5 MPa were closest to those of raw coal. He et al.^27^ reported that rock strength improved the deformation resistance of composite materials. Liu et al.^28^ suggested that the development of AE energy represented different stages of the cutting process. Xia et al.^29^ reported that the AE model exhibited an initial growth period, a stable growth period, and a rapid decline period. Li et al.^30^ reported a good positive correlation between the statistical parameters of acoustic emission signals and the water pressure curve. Zhang et al.^31^ suggested that the acoustic emission time series had fractal characteristics and that the correlation dimension characterized the degrees of damage to coal samples. Yin et al.^32^ reported that AE events exhibited peak fluctuations in the later stages after peak destruction (Supplementary file).

The research cited above indicated that the rock mass structure and loading method have significant impacts on acoustic emissions. However, coal rock masses exhibit complex mechanical behaviour and acoustic emission characteristics due to their complex structures and compositions. It is necessary to conduct in-depth research on the structural impacts of these materials on the acoustic emissions. Currently, research is limited mainly to the acoustic emission characteristics of gas-containing coal bodies, and relatively little research has been conducted on the acoustic emission characteristics of coal gas hydrate systems. Therefore, research on the impact of acoustic emissions from coal containing gas hydrates can be used to analyse the mechanical properties of coal containing gas hydrates, verify the ability of coal containing gas hydrates to improve outburst coal seams, and enrich and improve the theory of gas hydrate solidification and outburst prevention technology.

Based on this, this article takes coal bodies containing gas hydrates as the research object, describes acoustic emission experiments performed on coal bodies containing gas hydrates under different saturations and confining pressures, and combines the elastic, yield, and failure stages of deformation and failure of coal bodies containing gas hydrates to reveal the influence of gas hydrate formation and saturation on the acoustic characteristics of outburst coal bodies.

## Experiment

### Experimental device

**Figure 1:**
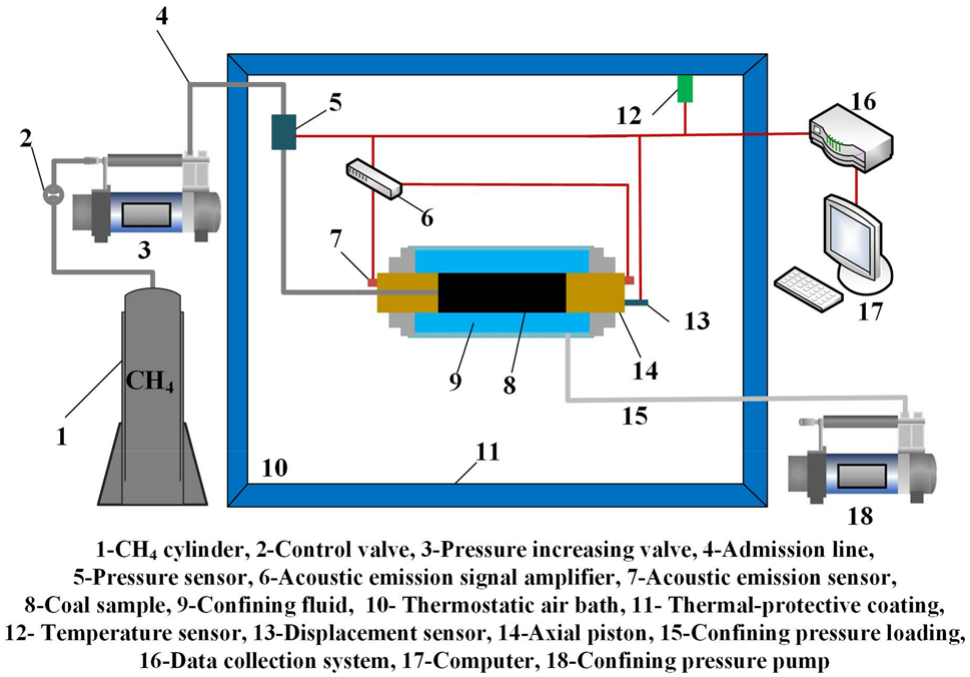
Experimental equipment.

An integrated experimental device for in situ generation and testing of the acoustic emissions from gas hydrates in coal was designed to study the acoustic emissions of coal containing gas hydrates. The device consisted of a multifunctional coal rock holder, a triaxial loading system, an acoustic emission signal acquisition system, a temperature control system, a gas pressure control system, and an experimental data acquisition system. This device generated gas hydrates in situ and obtained the acoustic emission characteristics of coal containing gas hydrates and featuring different crystal types, saturation, loading conditions, and additives. A diagram and schematic diagram of the equipment are shown in Fig. 1.

This study used an acoustic emission signal acquisition and analysis system for the SH-II all-weather health monitoring system produced by the American Physical Acoustics Company. The acoustic emission signal acquisition and analysis system mainly consisted of an acoustic emission probe, a preamplifier, a filter, a main amplifier, a computer, and the corresponding data processing and analysis software.

Both the fixed end of the axial pressure and the sample were solid pads, which facilitated the transmission of acoustic emission signals. Therefore, the acoustic emission probe (as shown in Fig. 1) was arranged at the fixed end where the axial pressure was applied. The acoustic emission probe model was an SR150M, and the dimensions were Ф19 × 15 mm, the frequency range was 60–400 kHz, the resonant frequency was 300 K, the weight was 22 g, the operating temperature range was − 20–120 °C, and the sensitivity peak was > 75 dB.

### Sample preparation

The mechanical properties of coal seams with coal and gas outbursts are poor, and the success rate of raw coal sampling is low. Even if hard blocks were prepared as samples, it was difficult to represent the properties of the entire coal seam. Yin et al.^33^ also reported that for specific mechanical parameters, such as compressive strength and elastic modulus, shaped coal and raw coal exhibited certain differences. However, in terms of the deformation parameters and compressive strength change rules, shaped coal and raw coal were consistent. Therefore, considering the poor mechanical properties of coal seams and gas outbursts, as well as the consistency between coal seams and raw coal seams in terms of regularity, coal was selected for research on the influence of saturation changes on the mechanical and acoustic emission characteristics of coal containing gas hydrates.

Therefore, in this experiment, a coal sample with good uniformity and a high preparation success rate was used as the sample, and the preparation process was as follows:

1. The coal samples taken underground were tightly wrapped with waterproof film, placed in the sample box and transported back to the laboratory to avoid changes in the moisture contents of the coal samples.
2. The coal sample was removed from the sample box and put into a pulveriser to break it into coal powder.
3. A certain amount of coal powder was put it into the screening machine, and it was thoroughly screened to obtain coal powder with particle sizes of 60–80 mesh.
4. A certain amount of 60–80 mesh coal powder and distilled water were added to the mixture, the stirring speed was set to 124 r/min, and the stirring duration was 15 min. The fully stirred coal powder was removed and put into the mould. The mould containing the coal powder was placed on the press, a force of 100 MPa was applied, and the process was continued for 30 min (a pressure of 100 MPa is commonly used for preparing coal, and the compressive strength of the coal prepared under this pressure was relatively close to that of the raw coal^34,35^); additionally, the compressed and formed coal powder sample measuring φ 50 × 100 mm was removed from the coal sample with nonparallelism of the end face not exceeding 0.05 mm for the next drying step.
5. The briquette sample was placed in a drying oven, the drying temperature was set to 50 °C, and the sample was weighed every 20 min. When the weight of the briquette approached the target weight, the weighing interval was shortened to ensure that the moisture in the briquette was at the target value. When the weight of the briquette reached the target weight, the briquette was removed, the briquette was tightly wrapped in cling film, and the briquette was directly placed in a triaxial chamber for acoustic emission experiments.

**Figure 2:**
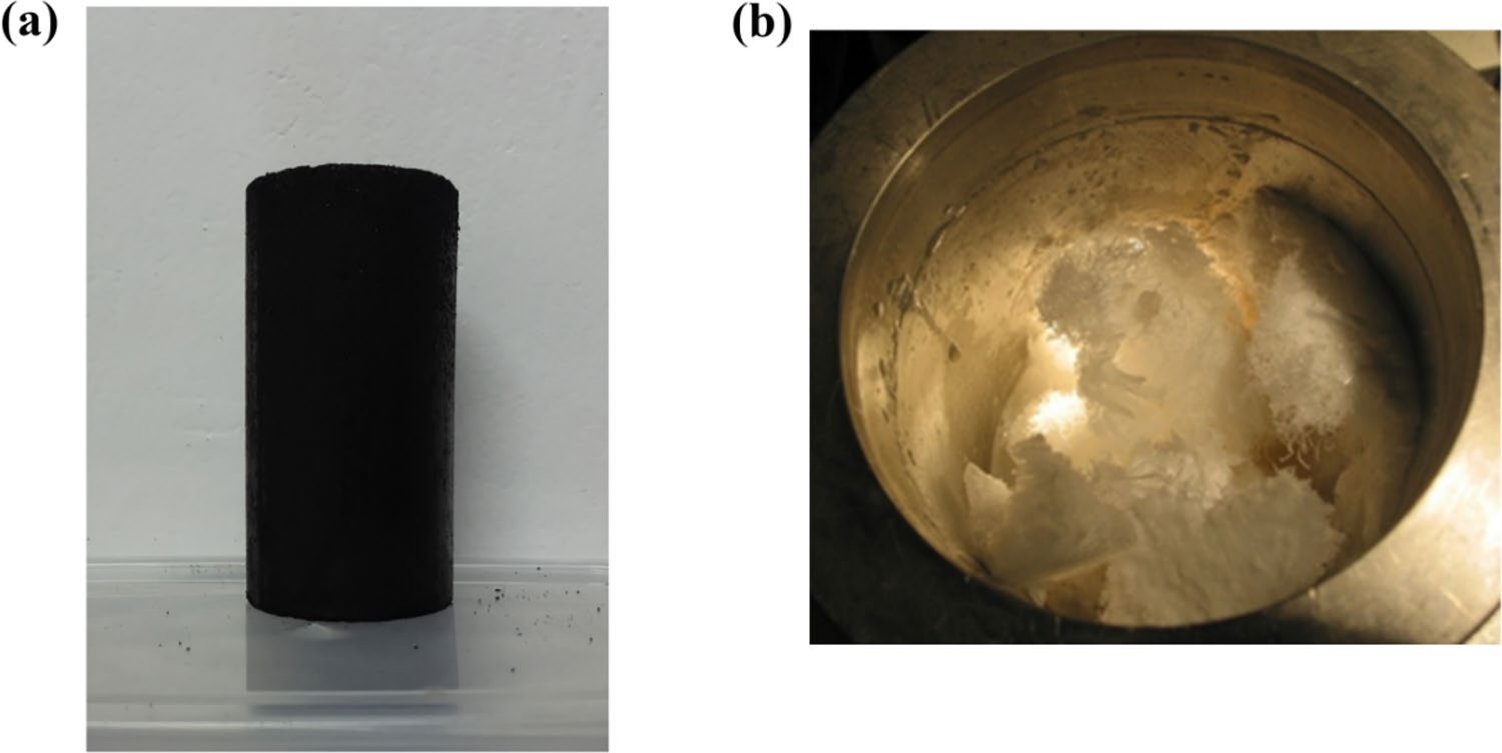
Coal sample and generated hydrates (**a**) Briquette sample, (**b**) Gas hydrate.

To approximate the properties of the samples to those of coal bodies at risk for coal and gas outbursts, an experimental coal sample was taken from a coal seam in the Xinan Coal Mine of the Longmei Group with a tendency to burst. Pure water was generated in-house in the laboratory. The methane gas used was purchased from Harbin Tongda Gas Co., Ltd. The gas sample composition was 99.99% CH_4_. Figure 2a shows the briquette sample, and Fig. 2b shows the gas hydrate generated in the experiment.

### Experimental procedure

Based on the acoustic emission characteristics of the coal containing gas hydrates, the effect of loading process saturation on the corresponding acoustic characteristic parameters was determined. Saturation is an important parameter for measuring the effects of hydrates on the acoustic characteristics of coal, and saturation control is a key technical challenge in this experiment.

At present, there are two main methods for saturation control. One is the water excess method. The experiment starts by injecting gas into the coal sample at once, then closing the valve and starting the hydration reaction. As the gas enters the hydrate cage in the gas phase space to form hydrates, the gas phase pressure gradually decreases. When the reaction stabilizes, the gas phase pressure remains unchanged. After a period of stability, the end of hydrate generation is determined based on the temperature and pressure at the beginning and end of the hydration reaction. Combined with the gas state equation, the hydrate saturation in the coal sample can be calculated. The second method is the gas excess method. During the hydration reaction, whenever the gas phase pressure decreases by a certain amount, the gas is replenished until the gas phase pressure no longer decreases. Assuming that the water in the coal sample reacts completely, the saturation level is calculated based on the initial water content.

Both saturation control methods have advantages and disadvantages. The saturation level calculated via the water excess method is relatively accurate, but because some water does not participate in the reaction, the final saturation is low; this limits the saturation setting range of the experiment, and the randomness of hydrate generation can also cause differences in the final saturation level under the same initial conditions. In the gas excess method, a small amount of bound water on the surface of the coal body does not participate in the hydration reaction, which decreases the actual saturation level to less than the calculated level; however, the difference in the saturation levels of coal samples with different gas hydrates under the same initial temperature and pressure decreases, facilitating analysis and discussion with more accurate test results.

In summary, due to the advantages of the gas excess method, such as small differences in saturation levels and wide saturation ranges among coal samples containing different gas hydrates, this study adopted the gas excess method to generate hydrates in the coal samples and investigated the influence of saturation factors on the acoustic emission characteristics of gas hydrated coal bodies^12^.

The calculation process of the initial water content required for a certain hydrate saturation is as follows:

The hydrate saturation level was the ratio of the hydrate volume to the total pore volume of coal and was calculated as follows:

1 $$ S_{h} = \frac{{V_{h} }}{{V_{m} }} \times 100\% $$

where *V*_*h*_ and *V*_*m*_ are the volume of the hydrates and the total pore volume of the coal samples, respectively.

According to Formula (1), for the same total volume of coal pores, different hydrate saturation levels correspond to different hydrate volumes. The total volume of the coal pores was calculated with Formula (2):

2 $$ V_{m} = m_{s} \times V_{g} $$

where ms is the mass of the briquette sample (260 g, empirical value), and the pore volume *V*_*g*_ is the average value of three test pore volumes for the same particle sizes.

Given target hydrate saturation levels of 20, 40, 60, and 80% with a known total pore size of the coal, the hydrate mass can be calculated with Formula (3):

3 $$ m_{h} = V_{h} \times \rho_{h} $$

where *V*_*h*_ is the hydrate volume, and ρh is the hydrate density. Due to the experimental conditions used in this study, the methane hydrate generated was generally type Ι. The density of the methane hydrate was assumed to be *ρ*_*h*_ = 0.91 g/cm^3^.

The hydration process of methane gas is represented with Eq. (4):

4 $$ {\text{8CH}}_{{4}} + {\text{46H}}_{{2}} {\text{O}} \leftrightarrow {\text{8CH}}_{{4}} \cdot {\text{46H}}_{{2}} {\text{O}} $$

5 $$ m_{w} = m_{h} \times (46 \times 18)/(46 \times 18 + 8 \times 16) $$

where *m*_*w*_ is the mass of water required to achieve the target saturation level.

Assuming that water fully participates in the reaction to generate hydrates, the initial water content required for a complete reaction under hydrate saturation conditions can be calculated with Formula (5). In the experiment, the aim of controlling the hydrate saturation level in the coal body was achieved by controlling the initial water content of the coal sample. The initial water contents are shown in Table 1.

**Table 1:** Initial water content result.

Particle size	Saturation%	Initial moisture content (g)	Hydrate mass (g)	Coal powder mass (g)
60 ~ 80 mesh	20	8.25	9.53	260
	40	16.49	19.04	
	60	24.74	28.56	
	80	32.98	38.08	

After the saturation level was controlled, the specific steps were as follows:

1. The prepared briquette sample was wrapped with the upper and lower plugs of the holder with a heat shrink tube, the sample was placed in the holder, the acoustic emission probe was installed at the fixed end of the axial pressure, a confining pressure of 0.5 MPa was slowly applied, 0.1 MPa of methane was introduced into the holder, and the sample was emptied three times to ensure that the air in the pipeline and holder was discharged.
2. A confining pressure of 5 MPa and a pressure of 4 MPa were applied for 12 h to dissolve the gas in the water. Axial pressure was applied at a speed of 0.01 mm/s until the gas-containing coal sample was damaged or the axial strain of the sample reached 15%, at which point the experiment ended. The temperature of the incubator was set to 0.5 °C, and cooling started. When the temperature was lower than the equilibrium temperature corresponding to the methane pressure, a pressure drop caused by hydrate formation occurred. When the pressure remained constant for 12 h, hydrate formation was considered to be complete, and the test of the acoustic emission characteristics of coal containing gas hydrate began.
3. A confining pressure and air pressure were applied at the target value, and axial pressure was applied at a speed of 0.01 mm/s until the coal containing gas hydrate was damaged or the axial strain of the sample reached 15%. The experiment was completed, and the experimental data were collected and analysed. The sample and experimental conditions were replaced for the next set of experiments. The experimental conditions are shown in Table 2.

**Table 2:** Experimental conditions for the influence of saturation on the acoustic emission characteristics of gas hydrate bearing coal.

Gas	Temperature/℃	Pressure/MPa	Confining pressure/MPa	Saturation/%
CH_4_	0.5	4	5	20
				40
				60
				80
			7	20
				40
				60
				80
			9	20
				40
				60
				80

## Experimental results and analyses

### Evolution of the acoustic emission characteristics of coal containing gas hydrates during triaxial loading

**Figure 3:**
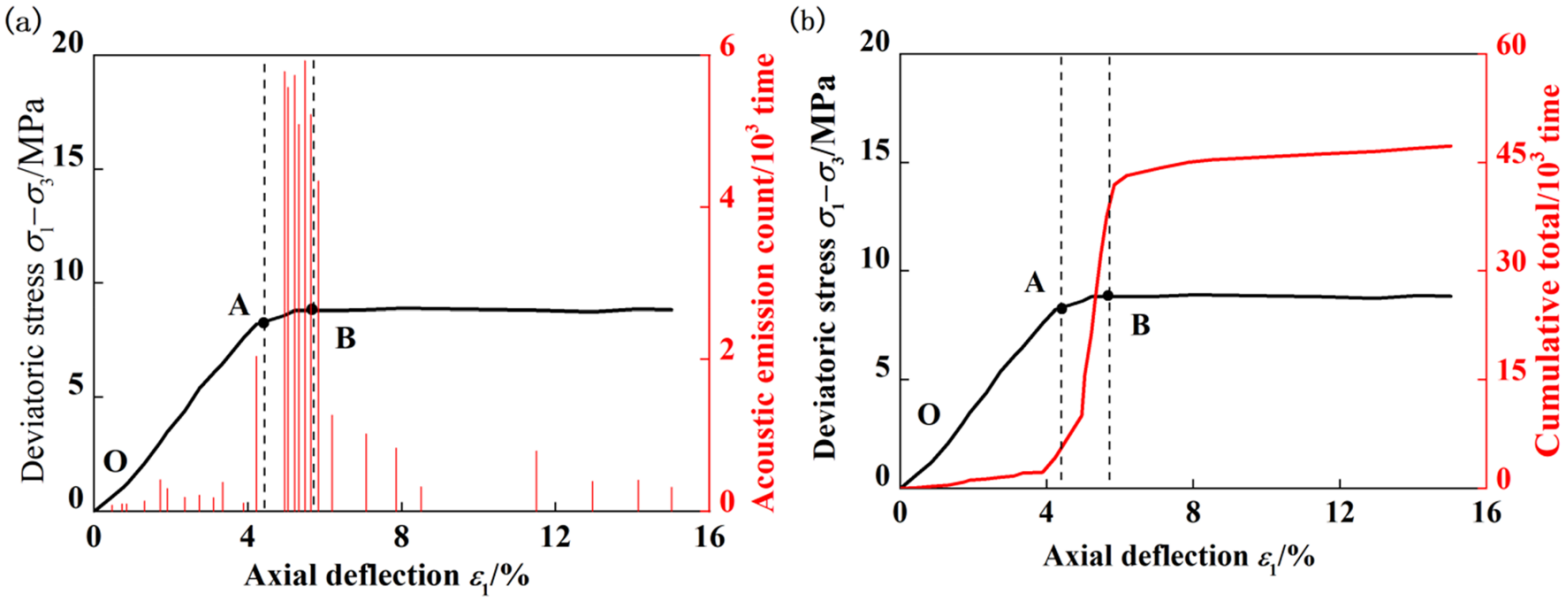
Change of acoustic emission ringing count of gas hydrate bearing coal during loading process. (**a**) Real time acoustic emission ringing count, (**b**) accumulated acoustic emission ringing count curve.

The acoustic emission ringing count refers to the number of oscillations that exceeded the threshold signal, and the cumulative acoustic emission ringing count refers to the cumulative value of the acoustic emission ringing count within a certain stage. The acoustic emission ringing count reflects the strengths and frequencies of the acoustic emission signals and is widely used in evaluations of acoustic emission activity^15^. Therefore, this article takes the acoustic emission ringing count as the characteristic parameter to determine the variation law for the acoustic emission characteristics of gas hydrate coal bodies. The changes in the acoustic emission ringing counts of the coal bodies containing gas hydrates under different confining pressures and saturation levels exhibited similar patterns. Therefore, the confining pressure was 5 MPa and the saturation level was 40%, for example, as shown in Fig. 3. As shown in the figure, the stress‒strain curve of coal containing gas hydrates exhibited strain hardening. There was a stage correlation between the acoustic emission ringing count and the stress responses of coal bodies containing gas hydrates. Elastic stage: The cumulative acoustic emission ringing count increased slowly, resulting in a small number of acoustic emission events. Yield stage: A sudden increase in the ringing count usually occurred before and after the yield point, followed by the detection of more intense acoustic emission activity, after which the acoustic emission ringing count remained high. Failure stage: There were peaks in the acoustic emission ringing count before and after the failure point, resulting in a certain amount of acoustic emission activity. During this stage, there were many acoustic emission events. The stress‒strain curves of coal bodies containing gas hydrates mainly consisted of an elastic stage, yield stage, and failure stage, with the characteristics of each stage as follows:

1. Elastic stage (OA stage): The relationship between the stress and strain was approximately linear, and compaction was not obvious. The cumulative acoustic emission ringing count increased slowly, resulting in a few acoustic emission events. The acoustic emission ringing count in the elastic stage accounted for only 8.97% of the total cumulative ringing count, indicating that elastic deformation occurred in the coal body in the early stage of loading, and the occurrence and propagation of cracks were less common. A small number of acoustic emission events came from the sliding friction of coal particles and the closure of microcracks. The elastic stage of coal containing the gas hydrate was relatively long, and its corresponding axial strain range was 0–4.95%.
2. Yield stage (AB stage): As the stress increased, the load borne by the coal containing gas hydrate gradually exceeded the elastic limit. The slope of the stress‒strain curve increased from point A, and the coal underwent irreversible plastic deformation. Sudden increases in ring count generally occurred before and after yield point A. After that, more intense acoustic emission activity was detected, and the acoustic emission ring count remained high. The cumulative ring count during the yield stage increased rapidly, with acoustic emission ring counts accounting for more than half of the total cumulative ring counts, 70.51%. During the yield stage, microcracks in the coal sample began to form and expand rapidly and unstably, marking a precursor to failure. The yield stage of coal containing gas hydrate was relatively short, and its corresponding axial strain range was 4.95%–5.83%.
3. Failure stage (after point B): As the stress further increased, there was a peak in the acoustic emission ringing before and after point B, and microcracks gradually developed into throughgoing cracks. After failure, the coal sample containing gas hydrate still maintained a certain bearing capacity, exhibited strain hardening, and generated a certain amount of acoustic emission activity. During this stage, there were many acoustic emission events, and the ringing count accounted for 20.52% of the total ringing count. The failure stage of coal containing gas hydrate was relatively long, and its corresponding axial strain range was 5.83%–15.02%.

### Influence of gas hydrate formation on the acoustic emission ring count of coal

**Figure 4:**
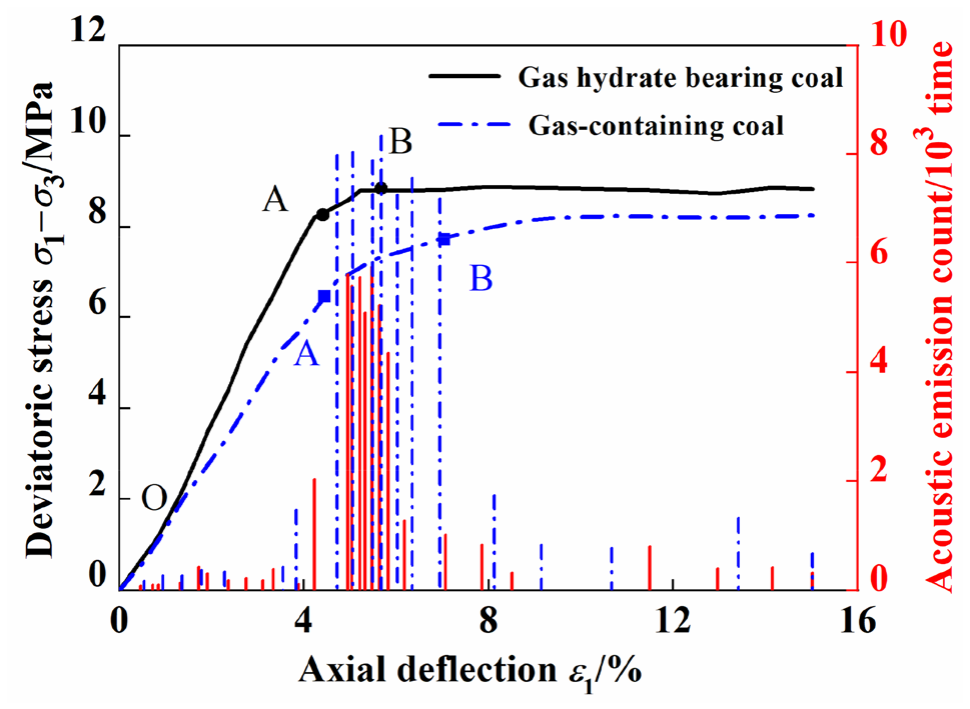
Acoustic emission ringing count during loading process of gas hydrate-bearing coal and gas-bearing coal under the same confining pressure.

Figure 4 shows the stress‒strain curves and acoustic emission ringing counts of coal containing gas and gas hydrates under a confining pressure of 5 MPa and a saturation level of 40%. The figure shows that in the elastic stage, the acoustic emission ringing values of gas containing coal and gas hydrate containing coal were relatively small, and the difference between the two was also relatively small. In the yield stage, the peak value of the acoustic emission ringing count for gas-containing coal was significantly greater than that for gas-containing hydrated coal, and the yield stage and high range of the acoustic emission ringing count for gas containing coal were both relatively long. During the failure stage, both gas-containing coal and gas hydrate-containing coal generated a few acoustic emission signals, and the acoustic emission ringing count of the gas-containing coal was greater than that of the gas hydrate-containing coal.

**Figure 5:**
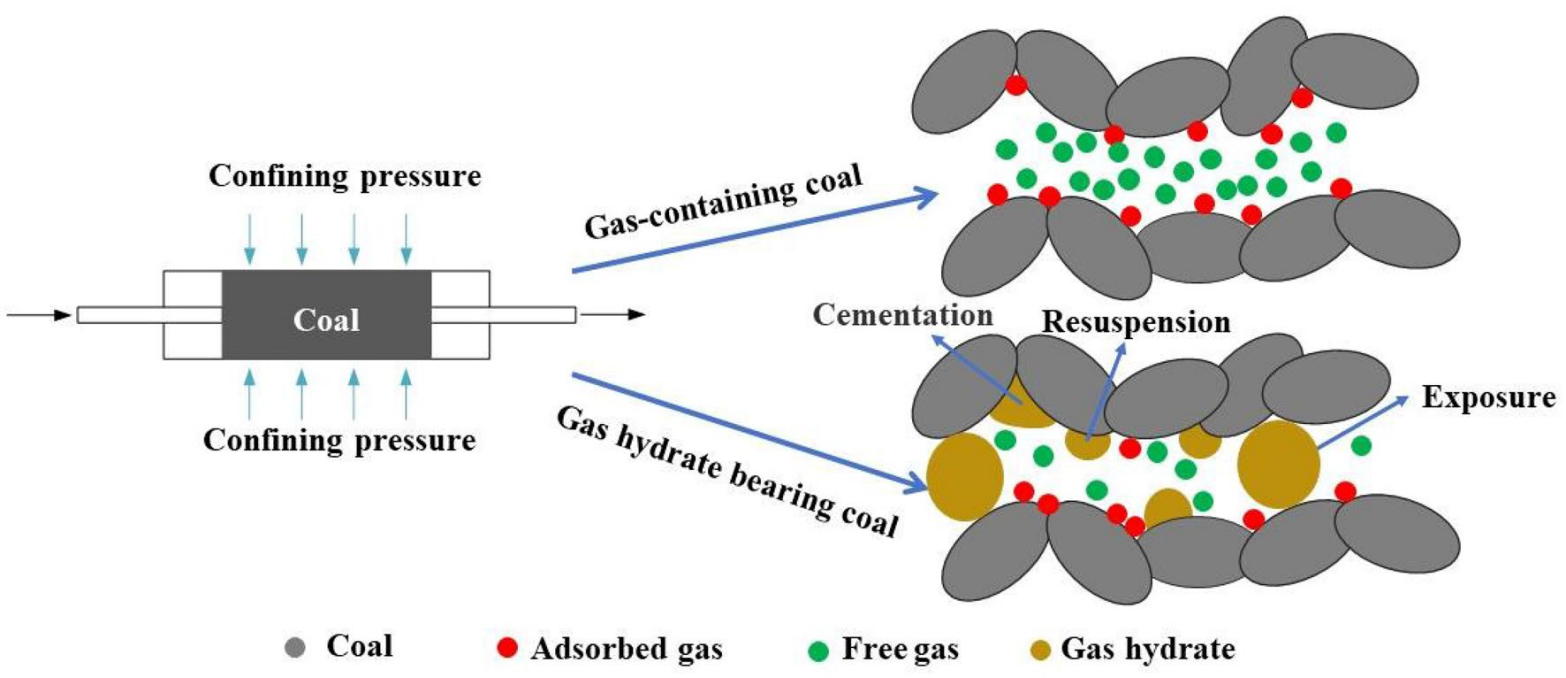
Schematic diagram of the influence mechanism of acoustic emission characteristics of coal bodies containing gas and gas hydrates.

Figure 4 indicates that during the loading process in the yield and failure stages, a larger acoustic emission ringing count indicated more severe sample failure. Under the same conditions, the coal containing gas hydrates exhibited lower acoustic emission ringing counts, which also indicated that gas hydrates were generated in the pore space of the coal, which the gas pressure and strengthened the mechanical properties of the coal. Therefore, under the same conditions, the coal containing gas hydrates exhibited lower acoustic emission ringing counts, demonstrating the impact of gas hydrate generation on the acoustic characteristics. The mechanism by which hydrates affected the acoustic emission characteristics of the coal containing gas hydrates is a key fundamental issue in the study of acoustic emission from coal containing gas hydrates. We believe that hydrates are generated in the pore space of coal, and their distribution pattern is an important factor affecting the acoustic emission characteristics of coal containing gas hydrates. There are three main distribution modes for hydrates in porous media: cementation, suspension, and contact^5^. Among them, cementation and contact are the main ways hydrates affect the acoustic emission characteristics of coal, as shown in Fig. 5. Hydrates are generated on the surfaces of coal particles and bind to the coal particles that were originally only in contact with each other and form a cohesive body, thereby improving the mechanical properties of the coal body and changing its original acoustic response. Hydrates are generated in the pore space of coal and come into contact with the coal particles that form the pore space. When the coal particles slide under external loads, the hydrates in contact with coal particles gradually bear some of the external load during the sliding process, resulting in an acoustic response that differs from that of gas-containing coal.

These experiments showed that gas hydrates had a strengthening effect on the mechanical properties of coal. The analysis suggested that hydrate particles played a role in cementation or adhesion of the coal, and the distribution pattern and possible influence mechanism of hydrates in coal pores should be investigated. We plan to use CT technology to directly observe the distribution patterns of hydrates in coal and elucidate the strengthening mechanisms of hydrates on the mechanical properties of coal through a combination of macroscopic and microscopic methods.

### Evolution of the acoustic emissions of coal bodies containing gas hydrates with different saturation levels

**Figure 6:**
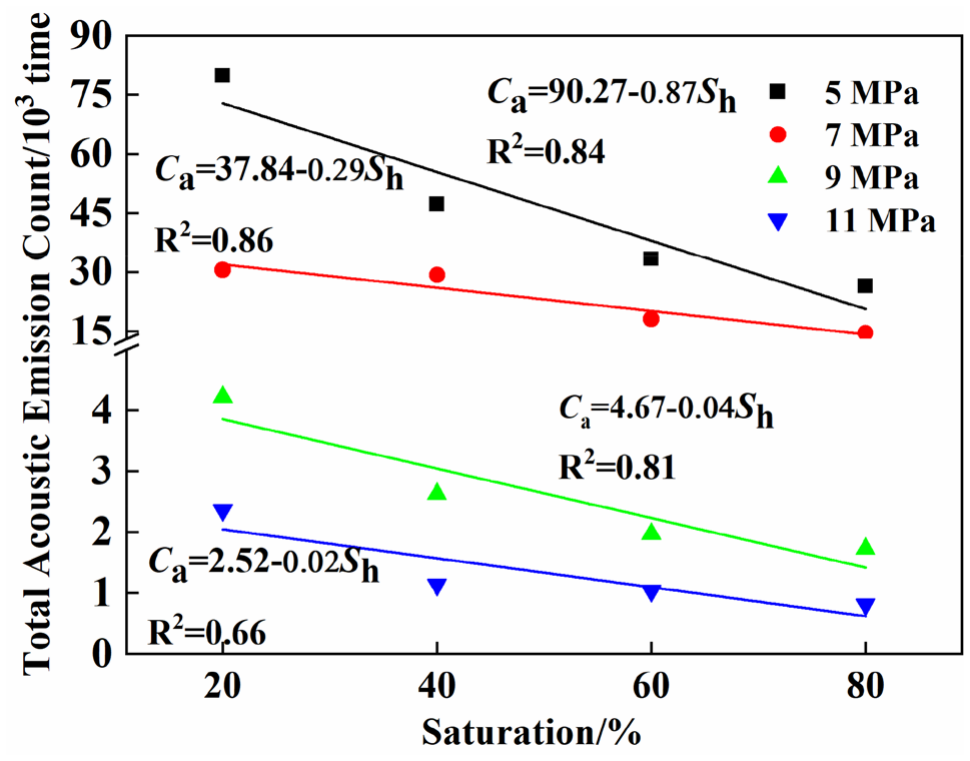
Cumulative acoustic emission ringing counts of gas hydrate bearing coal at different saturations.

The saturation level is the ratio of the hydrate volume to the total volume of coal pore space and is an important parameter for measuring the number of hydrates and the properties of a hydrate coal system^5^. Due to differences in the water volume and the randomness of hydrate generation, gas hydrate solidification and outburst prevention may produce gas hydrate coal bodies with significant saturation level differences. Therefore, to study the effect of saturation on the acoustic emissions of gas hydrate coal bodies during the entire triaxial loading process, Fig. 6 shows the cumulative acoustic emission ringing counts of coal bodies containing gas hydrates under different saturation levels and confining pressures. The figure shows that with the same confining pressure, the cumulative ringing count decreased approximately linearly with increasing saturation level. At a confining pressure of 7 MPa, the cumulative ringing count decreased the most, with saturation increasing from 40 to 80%. The cumulative ringing count decreased to the original value of 0.57, while at confining pressures of 5 MPa and 9 MPa, it decreased to the original values of 0.46 and 0.34, respectively. As the confining pressure increased, the effect of the saturation level on the cumulative ringing count of the coal containing gas hydrates decreased, and the slope of the fitted curve showed a significant decreasing trend, indicating that an increase in the confining pressure limited the impact of changes in the hydrate content on the acoustic emission ringing count of the coal.

By fitting the cumulative acoustic emission ringing counts of coal bodies containing gas hydrates under the same confining pressures and different saturation levels, the fitting curve of the cumulative acoustic emission ringing counts of coal bodies containing gas hydrates in Fig. 5 was obtained. The fitting equations under confining pressures of 5, 7, 9, and 11 MPa were Ca = 90.27–0.87*S*_h_, Ca = 37.84–0.29*S*_h_*,* Ca = 4.67–0.04*S*_h_, and Ca = 2.52–0.02*S*_h_, respectively, and the R^2^ values for the fits were 0.84, 0.86, 0.81, and 0.66, respectively. Due to the difficulty and complexity of conducting indoor experiments, the data obtained from these experiments are generally limited by the small number of experimental points. To enhance the accuracy of the experimental data in predicting on-site conditions, a fitting method was used to obtain the relationship between the cumulative acoustic emission ringing count and saturation level. Based on the fitting relationship, the cumulative acoustic emission ringing count at a certain point within the experimental condition range was determined, providing basic and experimental parameters for gas solidification and outburst prevention technology. Among the three confining pressures, the fitting degree was closest to 1 at a confining pressure of 7 MPa, and the fitting effect was the best.

### Influence of saturation on the cumulative acoustic emission ringing counts of coal bodies containing gas hydrates at different stages

**Figure 7:**
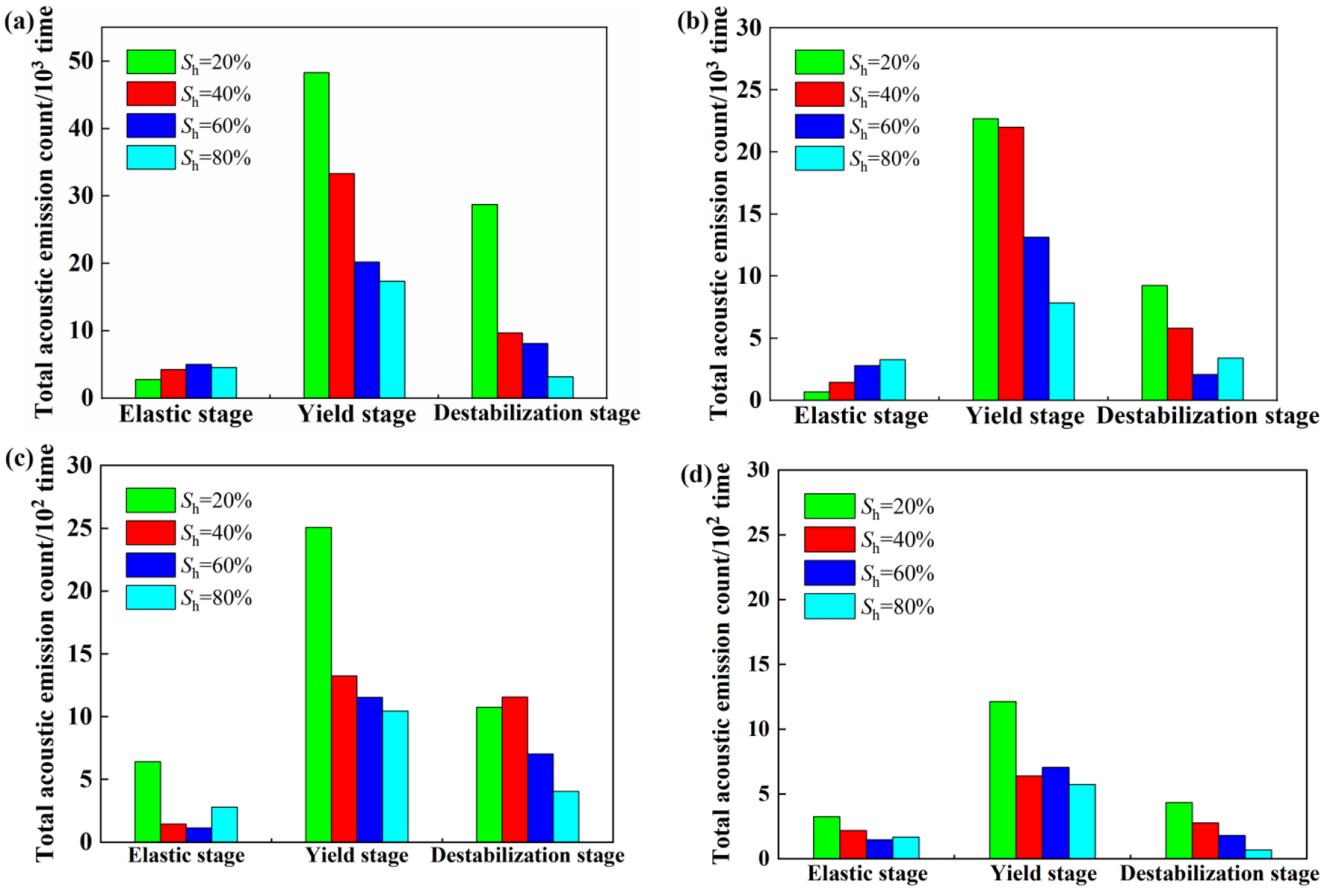
Influence of saturation on accumulative acoustic emission ringing count of gas hydrate bearing coal in different stages (**a**) 5MPa, (**b**) 7MPa, (**c**) 9MPa, (**d**) 11MPa.

Figure 3 shows that the acoustic emission ringing counts of the coal samples containing gas hydrates exhibit different evolution patterns at different stress and strain stages, while Fig. 6 shows that saturation was the main factor affecting the total cumulative acoustic emission ringing counts of the coal samples containing tile hydrates. Therefore, to study the effect of saturation on the stress‒strain acoustic emissions of coal containing gas hydrates at various stages, their cumulative acoustic emission ringing counts were calculated for different saturation levels during the loading process. Combined with the range of deformation and failure stages, Fig. 7 shows the cumulative acoustic emission ringing counts during the elastic, yield, and failure stages. As shown in the figure, there were more cumulative acoustic emission ringing events in the yield stage, fewer cumulative ringing events in the failure stage, and the fewest cumulative ringing events in the elastic stage. Under the same confining pressure, there was a small difference in the cumulative ringing counts of coal samples containing gas hydrates with different saturation levels in the elastic stage. The maximum difference in the cumulative ringing counts among the three saturation ranges was 0.78 × 103. Increases in the number of hydrates in the pores of the coal body had a relatively small impact on the acoustic emission activity generated by compaction and slight slip during the elastic stage of the coal body. An analysis revealed that the coal sample exhibited elastic deformation, with only slight slip, resulting in a few acoustic emission events, while hydrates were distributed in the pores of the coal body in the filling, wrapping, and cementation states. During the elastic stage, slight slip had a small impact on the interactions between hydrates and the coal sample skeleton particles.

In the yield stage, saturation had a significant impact on the cumulative acoustic emission ringing counts of the coal samples. The cumulative acoustic emission ringing counts of coal samples containing gas hydrates decreased with increasing saturation levels. After the saturation level increased from 40 to 80%, the cumulative acoustic emission ringing counts of the coal samples containing gas hydrates in the yield stage decreased to the original values of 0.49 (confining pressure 5 MPa), 0.64 (confining pressure 5 MPa), and 0.21 (confining pressure 5 MPa). Increases in the amounts of hydrates in the pores of the coal samples suppressed the slippage of particles and even fracture surfaces during the yield stage, and the cumulative ringing counts of the coal samples with gas hydrates at higher saturation levels were relatively low during the yield stage. An analysis suggested that when the coal sample entered the yield stage, irreversible plastic deformation occurred, the coal pore space was compressed, and microcracks gradually developed. The sliding activities of particles and fracture surfaces became intense, and the distribution of hydrates between coal particles played a role in cementing the coal particles or supporting the pore space, inhibiting the sliding of particles and fracture surfaces that generate acoustic emission activity, thereby causing the above phenomena^22^.

During the failure stage, saturation had an impact on the cumulative ringing counts of the coal samples containing gas hydrates, and the cumulative ringing count decreased with increasing saturation levels. Under a confining pressure of 9 MPa, as the saturation increased, the cumulative ringing count decreased the most, by 64.92%. This indicated that changes in the hydrate content had an impact on the acoustic emission activity during the failure stage of gas-containing hydrated coal bodies. An analysis suggested that the stress‒strain curves of the coal containing gas hydrates mostly exhibit a strain hardening type, and there was no obvious stress drop. That is, during the failure stage, as the axial load was applied, the stress that the coal containing gas hydrates bore barely changed. This may be because the allowable stress level of the coal containing gas hydrates during the failure stage mainly came from slippage of the connecting surface. However, during the slip process of the connecting surfaces, the cementation or support effect of the hydrates still played a certain role, so saturation had an impact on the cumulative ringing counts of gas hydrate coal bodies during the failure stage^16^.

### Effect of confining pressure on the acoustic emission ringing count of coal containing gas hydrates

**Figure 8:**
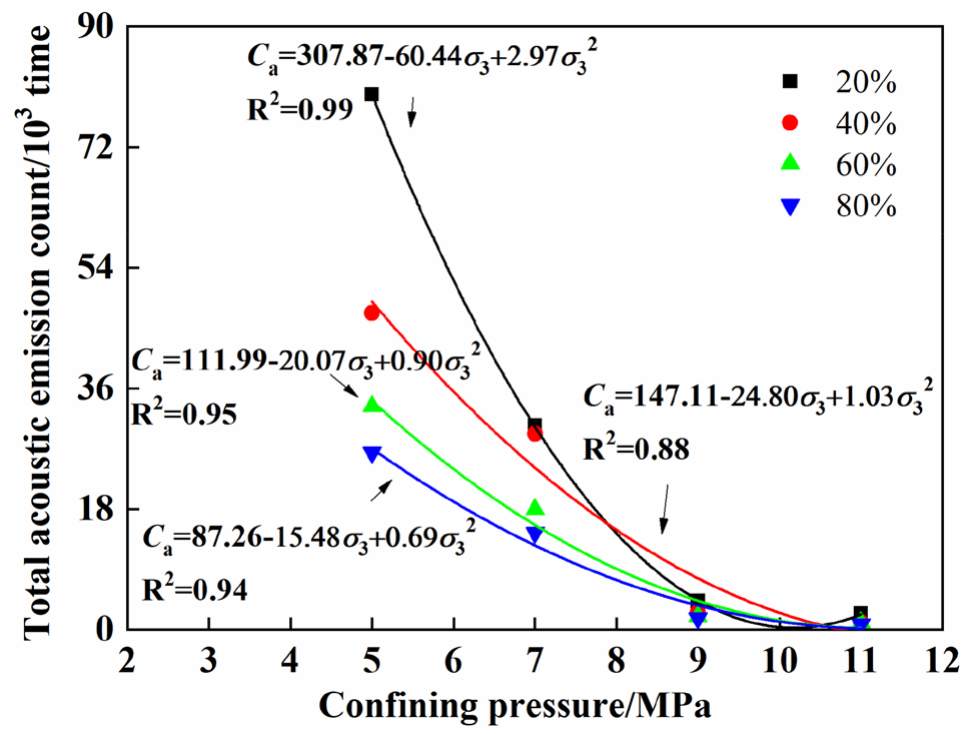
Cumulative acoustic emission ringing counts of gas hydrate bearing coal at different confining pressure.

In actual engineering, the coal seam is in a three-way stress environment, and the ground stress of the coal seam is closely related to the burial depth and tectonic movement. Therefore, to study the influence of confining pressure (ground stress) on the acoustic emission characteristics of gas hydrate coal during the whole process of triaxial loading, Fig. 8 shows the cumulative acoustic emission ringing count of gas hydrate coal under different confining pressures and degrees of saturation. The figure shows that under the same saturation level, the cumulative ringing count decreased in an approximately binomial manner with increasing confining pressure, and the decrease was significant. When the confining pressure increased from 5 to 11 MPa, the cumulative ringing count decreased by 97.05% (20% saturation), 97.60% (40% saturation), 96.91% (60% saturation), and 96.94% (80% saturation). The difference in the decreases in cumulative ringing counts at the different saturation levels was small. An increase in the confining pressure suppressed the acoustic emission activities of the coal samples containing gas hydrates during the loading process, and the extents to which the confining pressure suppressed acoustic emission from the coal samples containing gas hydrates and different saturation levels were relatively similar.

**Figure 9:**
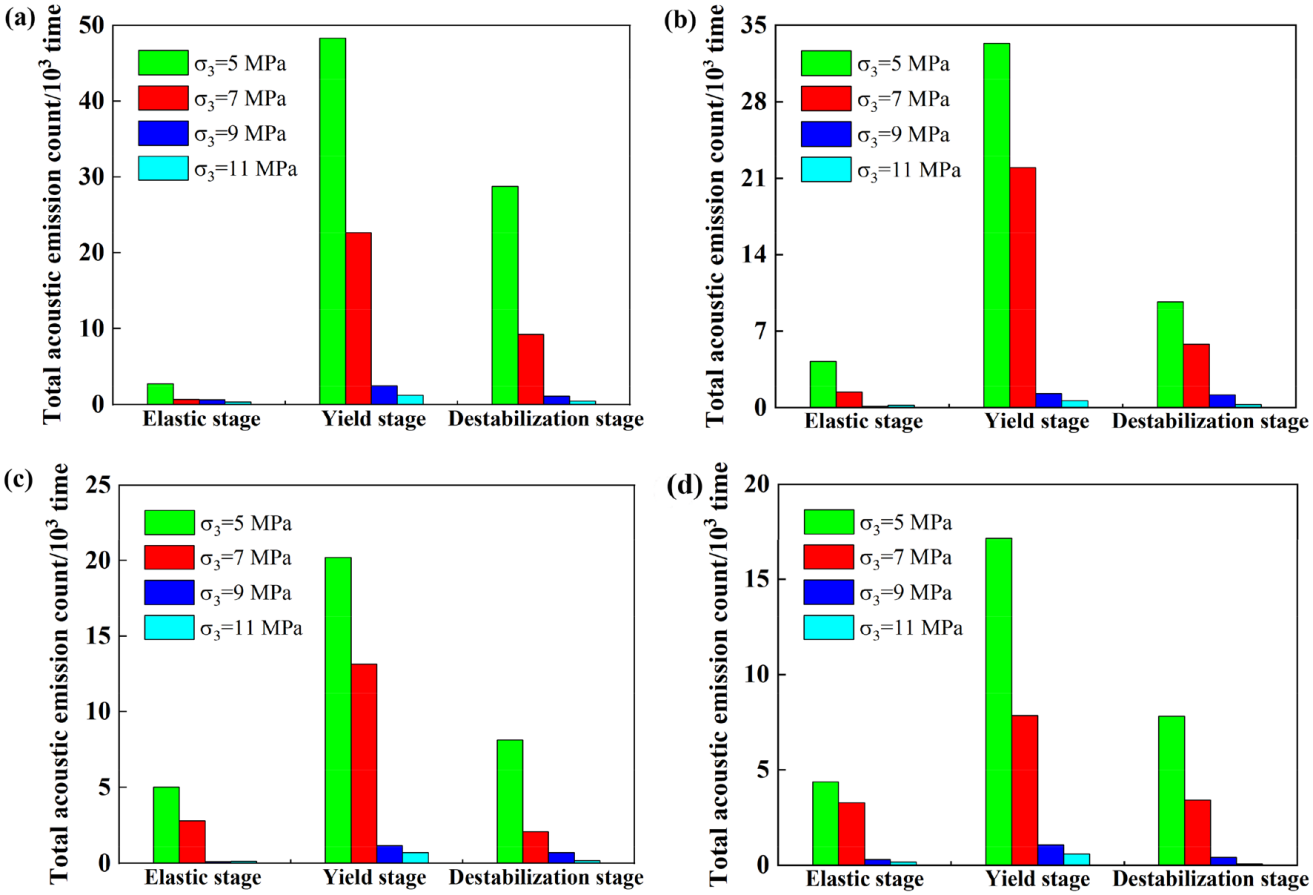
Influence of confining pressure on accumulative acoustic emission ringing count of gas hydrate bearing coal in different stages (**a**) Saturation 20%, (**b**) Saturation 40%, (**c**) Saturation 60%, (**d**) Saturation 80%

The cumulative acoustic emission ringing at different saturation levels during the loading process, combined with the range of deformation and failure stages, are shown in Fig. 9 for cumulative acoustic emission ringing counts of the gas hydrates containing coal at different confining pressures during the elastic, yield, and failure stages. As shown in the figure, at the same saturation levels, the cumulative ringing counts in the elastic stage, yield stage, and failure stage all decreased with increasing confining pressure. Under a confining pressure of 9 MPa, the cumulative ringing counts of the coal samples containing gas hydrates at each stage were the smallest, and compared to those at confining pressures of 5 MPa and 7 MPa, the decreases were significant. The influence of confining pressure on the cumulative ringing count of gas hydrate-containing coal at different stages under different saturation levels was similar. The inhibitory effect of confining pressure on the acoustic emission activities of coal bodies containing gas hydrates was reflected in various stages of stress‒strain, and this inhibitory effect was less affected by the saturation level. An analysis suggested that under the same axial pressure, a greater confining pressure limited the relative slip of the fracture surface, resulting in less acoustic emission activity and fewer acoustic emission rings.

## Conclusion

Acoustic emission experiments were conducted on coal containing gas hydrates at different saturation levels, combined with the elastic, yield, and failure stages of deformation and failure of coal containing gas hydrates, to reveal the influence of gas hydrate formation and saturation on the acoustic characteristics of outburst coal.

1. During the yielding and failure stages, the acoustic emission ringing count of gas-containing coal was higher than that of gas-containing hydrate coal.
2. There was a stage correlation between the acoustic emission ringing counts and the stress responses of coal bodies containing gas hydrates. Elastic stage: The cumulative acoustic emission ringing count increased slowly, resulting in few acoustic emission events. Yield stage: Sudden increases in the ringing count usually occurred before and after the yield point, followed by the detection of more intense acoustic emission activity, after which the acoustic emission ringing count remained high. Failure stage: There were peaks in the acoustic emission ringing counts before and after the destruction point, resulting in a certain amount of acoustic emission activity. During this stage, there were many acoustic emission events.
3. The cumulative ringing count of the coal containing gas hydrates showed an approximately linear decreasing trend with increasing saturation. Saturation had a relatively small impact on the cumulative ringing count during the elastic stages of coal samples containing gas hydrates, while the cumulative ringing count during the yield and failure stages of coal samples containing gas hydrates with different saturations varied significantly.
4. The confining pressure had an inhibitory effect on the acoustic emission activities of coal samples containing gas hydrates. As the confining pressure increased, the total cumulative ringing count and the cumulative ringing count at each stage of the coal sample containing gas hydrate decreased.

In subsequent research, the acoustic emission characteristics of coal samples containing gas hydrates under high stress should be investigated for better alignment with existing deep mining applications.

Due to the different physical signalling mechanisms generated by coal rock fractures, a single indicator rarely reflects the characteristics of coal failure comprehensively and accurately. Subsequent research should use a comprehensive indicator method comprising acoustic emission, electromagnetic induction signals, and other indicators to replace the single indicator method and achieve a more comprehensive and accurate reflection of coal failure characteristics.

### Supplementary Information

Supplementary Information.
